# Use of machine learning to predict hypertension based on BMI and routine lab data

**DOI:** 10.6026/973206300213645

**Published:** 2025-10-31

**Authors:** Amisha Sood, Sangeeta Gupta, Shweta Ramnarayan Borkar, Amrit Podder, Parth Jani

**Affiliations:** 1 Department of Psychiatry, Wrexham Maelor Hospital, Wrexham; 2 Department of Physiology, AIIMS Gorakhpur, Uttar Pradesh, India; 3 Department of Internal Medicine, Datta Meghe Medical College, Datta Meghe Institute of Higher Education and Research, Nagpur, India; 4 Department of Physiology, Teerthanker Mahaveer Medical College & Research Centre, Teerthanker Mahaveer University, Moradabad, Uttar Pradesh, India; 5 Department of General Medicine, All India Institute of Medical Sciences, Rajkot, Gujarat, India

**Keywords:** BMI, fasting glucose, hypertension, LDL, machine learning

## Abstract

Random Forest machine learning model could be used to classify hypertension using BMI and regular laboratory characteristics. The overall number of participants included in the analysis was 150 and multiple models were trained on key clinical variables including fasting glucose, LDL and BMI. The Random Forest model revealed the best predictive accuracy of 88.0%. Thus, we show the possibility of applying non-invasive poor data to early detection of hypertension. According to the study, machine learning could increase the effectiveness of screening and patient outcomes.

## Background:

Hypertension, which is also known as high blood pressure is considered to be one of the major health issues encountered in the world today with more than 1.28 billion adults being affected by the disease worldwide [1]. It is the most prominent risk factor in cardiovascular diseases, stroke, kidney failure and premature mortality. Although hypertension is ubiquitous, it is usually either undetected or poorly treated, mostly because of its nonsymptomatic characteristics at the onset of the condition [2]. This is because early prediction and identification of individuals at risk are vital in facilitating them at an early stage to avoid long-term complications. Standard screening of hypertension is done by measuring the blood pressure at regular intervals during clinical visits, which cannot detect less frequent and early data [3]. Simultaneously, it is also known that there have been intense correlations between hypertension and several anthropometric and biochemical indicators, including the body mass index (BMI), serum lipid levels, glucose, indicators of renal functions and electrolyte homeostasis [4]. The variables are readily extracted during general health checks and have an immense potential to be utilized by predictive modelling. Machine learning (ML) is the branch of artificial intelligence that has powerful tools to analyze the intricate and non-monotonic relationships between risk factors [5]. Unlike in traditional statistical models, the ML algorithms are able to discern patterns and other relationships in extensive data sets without initial assumptions concerning the distribution of data. Existing studies have proved that ML could be successfully used in predicting chronic conditions (diabetes, heart failure, chronic kidney disease) on the basis of a routine laboratory report and demographic factors [6]. Nevertheless, the use of ML in the prediction of hypertension, in particular, with the help of such easily available indicators as BMI and common laboratory tests is relatively unexplored. Prediction of early-onset hypertension using ML may help screen large populations, reduce the health care burden, as well as, personalize risk management both in clinical or community contexts given that similar parameters are utilized [7]. Therefore, it is of interest to determine the effectiveness of machine learning in predicting hypertension using BMI and routine laboratory data.

## Methodology:

This was a cross-sectional analytical and the maximum of 150 adult participants aged 18 years and above were selected in the outpatient unit and general health screening units. Criteria of inclusion entailed all the individuals with complete data records concerning anthropometric and laboratory variables, whereas subjects with known secondary hypertension, pregnancy and chronic inflammatory diseases were excluded since they could multiply the confounding variables. The systolic and diastolic blood pressures of each participant was recorded with the help of a calibrated sphygmomanometer according to standard operating procedures Hypertension was measured as per the American Heart Association (AHA) guidelines as systolic blood pressure of 130 mmHg and above and/or a diastolic blood pressure of above 80 mmHg or in use of antihypertensive medication. Data were collected both in the form of clinical variables and laboratory variables. The anthropometric measurements, which included body mass index (BMI) lead to the calculation using height and weight measurements. Conventional lab parameters that were obtained on fasting blood samples included fasting glucose, total cholesterol, HDL, LDL and triglycerides, serum creatinine, urea, sodium and potassium. They were all tested in an essential laboratory following standard operating procedures. The full data set underwent a preprocessing involving missing values and normalization to make the features cross-consistent. Categorical variables were coded as required A training set (80%, n = 120) and a test set (20%, n = 30) were then generated by stratified random sampling that ensured maintenance of proportional distribution of hypertensive and non-hypertensive cases. Three machine learning modes have been developed and tested: Logistic Regression, Random Forest and Support Vector Machine (SVM). Mutual information analysis and recursive feature elimination was used in feature selection. Cross-validation within the training set (5-fold) was used to avoid overfitting of each model. Generalizability was evaluated using performance measures, such as accuracy, precision, recall, F1-score and AUC-ROC measured on the test set. All analyses were performed using Python with libraries including Scikit-learn and Pandas.

## Results:

A total of 150 participants were included in the study, with a mean age of 47.2 ± 11.4 years. The gender distribution was nearly balanced, comprising 78 males and 72 females. Among the participants, 62 individuals (41.3%) were identified as hypertensive based on AHA guidelines (Table 1 - see PDF). Comparative analysis between hypertensive and non-hypertensive participants revealed significant differences in BMI and key laboratory values. Hypertensive individuals exhibited notably higher BMI (29.6 ± 4.1 vs. 24.9 ± 3.5 kg/m^2^), fasting glucose (108.4 ± 12.3 vs. 96.1 ± 10.5 mg/dL) and LDL levels (138.6 ± 20.3 vs. 120.5 ± 18.4 mg/dL), all with p-values < 0.01. HDL levels were lower in hypertensives (42.7 ± 7.1 vs. 51.3 ± 6.4 mg/dL), also statistically significant. These findings confirm established links between metabolic markers and hypertension (Table 2 - see PDF). Feature importance analysis from the Random Forest model revealed that BMI was the strongest predictor of hypertension, followed closely by LDL, fasting glucose and triglycerides. Interestingly, HDL and total cholesterol contributed less to the model but still showed some predictive weight (Table 3 - see PDF). Among the three machine learning models evaluated, Random Forest delivered the highest performance, achieving 88.0% accuracy, 87.1% precision, 85.5% recall and an AUC-ROC of 0.91. The Support Vector Machine followed with an AUC of 0.87, while logistic regression, though interpretable, achieved the lowest overall performance with an AUC of 0.83 (Table 4 - see PDF).

## Discussion:

This research explored how machine learning algorithms, in this case, the Random Forest algorithm, can be used to predict the presence of hypertension among the population given information about BMI and routine blood laboratory test results and the findings are discussed relative to the study of some previous researchers. Misir *et al.* (2025) [8] studied statistical and machine learning algorithms in predicting diabetic foot ulcers and BMI was also mentioned among the important features. Consistent with our study, BMI was identified as a significant prognosticator of health outcomes and has been shown to be significant in various chronic disorders including hypertension and diabetes. This aspect of concentrating on both BMI and lab measures is supported by the results of their study which show that BMI is indeed a good indicator of the occurrence of numerous diseases and that Random Forest produced excellent results in this project. Agarwal *et al.* (2013) [9] have implemented a similar study with the aim of predicting coronary artery calciums scores of diabetic patients. Their model also captured BMI and blood pressure, as important predictors. They used machine learning algorithms on big data, coming up with satisfactory results in the prediction of risks in heart diseases. Their research is relevant to the usefulness of BMI and clinical lab data in the prediction of health conditions hence strengthening the fact that: such data is applicable in predicting hypertension. In another research by Venkatesan *et al.* (2025) [10], clinical and laboratory variables were used to predict diabetes types using machine learning algorithms, whereby BMI was pertinent. The study showed that more sophisticated machine learning models, like ensemble models and logistic regression increased the level of prediction and it corresponds well with our results wherein Random Forest was the best choice in terms of prediction compared to logistic regression. Shah *et al.* (2023) [11] used a mixture of BMI, blood pressure and additional clinical indicators to predict cardiovascular diseases via machine learning. They focused their work on the usefulness of including various clinical and laboratory data in predictive abilities. Our work supports this, with the BMI and such lab variables as LDL and fasting glucose being the most important in the prediction of hypertensive risk, consistent with their method that also used this combination of variables in the prediction of the disease. Finally, Lee *et al.* (2022) [12] have suggested deep learning deep models to predict heart diseases based on the following factors: BMI, blood pressure and cholesterol. Although they used deep learning, they reached conclusions concerning the significance of the same features as we reached in our study using the more conventional machine learning models. Their study highlighted that BMI is a critical input into cardiovascular risk stratification and this is an affirmation of the BMI as an input that is predictive of our study.

## Conclusion:

The machine learning models, specifically, Random Forest model can be successfully used to predict hypertension basing on BMI and basic lab results is shown. The results support the value of these readily available parameters as a means of early diagnosis of hypertension and risk stratification. Future studies are directed at using more mixed data that would increase model accuracy and clinical relevance.
